# Correction: Inkjet assisted fabrication of planar biocompatible memristors

**DOI:** 10.1039/d0ra90019b

**Published:** 2020-03-09

**Authors:** Georgii A. Illarionov, Denis S. Kolchanov, Ivan Mukhin, Oleg A. Kuchur, Mikhail V. Zhukov, Ekaterina Sergeeva, Vladimir V. Krishtop, Alexandr V. Vinogradov, Maxim I. Morozov

**Affiliations:** Laboratory of Solution Chemistry of Advanced Materials and Technologies, ITMO University Lomonosova str. 9 St. Petersburg 191002 Russia maximm@alumni.ntnu.no morozov@scamt-itmo.ru; St. Petersburg Academic University Khlopina 8/3 St. Petersburg Russia; Institute for Analytical Instrumentation RAS Ivan Chernykh str. 31-33 St. Petersburg 198095 Russia; Research Center, Ivanovo State Medical Academy Sheremetevsky ave. 8 Ivanovo 153012 Russia

## Abstract

Correction for ‘Inkjet assisted fabrication of planar biocompatible memristors’ by Georgii A. Illarionov *et al.*, *RSC Adv.*, 2019, **9**, 35998–36004.

The authors regret that one of the co-authors, Ivan Mukhin, was omitted from the original article. The correct author list is as shown here.

The Royal Society of Chemistry apologises for these errors and any consequent inconvenience to authors and readers.

## Supplementary Material

